# Peri-arterial Autonomic Innervation of the Human Ear

**DOI:** 10.1038/s41598-018-29839-z

**Published:** 2018-07-31

**Authors:** Yusuf Ozgur Cakmak, Sebastian Cotofana, Carsten Jäger, Markus Morawski, Mircea-Constantin Sora, Michael Werner, Niels Hammer

**Affiliations:** 1 Department of Anatomy, School of Biomedical Sciences, University of Otago, Dunedin, New Zealand; 2 Albany Medical College, Department of Anatomy, Albany, NY USA; 3 University of Leipzig, Paul-Flechsig-Institute for Brain Research, Leipzig, Germany; 4 Sigmund-Freud Private University Vienna, Centre for Anatomy and Molecular Medicine, Vienna, Austria; 5 Medical University of Vienna, Zentrum für Anatomie und Zellbiologie, Vienna, Austria; 6 Fraunhofer Institute for Machine Tools and Forming Technology, Dresden, Germany; 7 Department of Trauma, Orthopedic and Plastic Surgery, University Hospital of Leipzig, Leipzig, Germany; 8 Brain Health Research Centre, Dunedin, New Zealand; 9 Medical Technologies Centre of Research Excellence, Auckland, New Zealand

## Abstract

Auricular vasomotor responses are considered to be signs of clinical conditions including migraine. The mechanisms of auricular vasomotor control are still debatable. This study aimed at investigating perivascular co-transmitters of vasomotor control in the auricle. Another aim was to provide three-dimensional arterial maps of the auricle, as a proxy of periarterial autonomic innervation. Twelve paired human auricles were used to visualize the arteries following Spalteholz clearing and *μ*-CT-based reconstruction. Perivascular innervation staining was conducted using anti-tyrosine hydroxylase (TH), anti-neuropeptide Y (NPY), anti-vasoactive intestinal peptide (VIP) and anti-choline acetyl transferase (ChAT). The combined Spalteholz technique and *μ*-CT revealed a highly consistent arrangement of the auricular vasculature. The superficial temporal (STA) and posterior auricular artery (PAA) supply the helical rim arcade and arcade, with the STA mainly forming the superior and the PAA forming the middle and inferior auricular artery. Co-existence of sympathetic NPY+ and TH+ terminals mediating vasoconstriction, and VIP+ and ACh+ indicating cholinergic vasodilatation, was found in the perivascular zone. The presence of both sympathetic vasoconstriction and cholinergic co-innervation for active vasodilatation was shown in the perivascular auricular zone. Assuming that the highly-consistent vasculature gives way to these terminals, this periarterial innervation may be found spread out across the helix.

## Introduction

The blood supply of the human auricle is known to be provided by the branches of both the superficial temporal artery (STA) and the posterior auricular artery (PAA), both being part of the circulation of the external carotid artery^1,2^. The predominant mechanism of arterial vasomotor control is based on changes of the sympathetic vascular tone. Vasodilation has been conclusively shown to be the effect of inhibiting the sympathetic vasoconstrictor tone but not due to increased activity of parasympathetic influences^3^. It has also been postulated that facial nerve parasympathetic fibers may be responsible for the facial autonomic symptomatology on the basis of the trigemino-autonomic reflex (TAC) hypothesis, to the effect that may cause vasodilatation of the ear vasculature^4^. Prominent trigemino-parasympathetic activations have been reported in case of TAC, along with sympathetic deficits^5^.

These vasoregulatory mechanisms may have implications for a few signs and symptoms related to the auricle. The red ear syndrome (RES) is known to correlate with migraine, upper cervical disorders and with trigeminal autonomic cephalgia^6–15^. In the context of the interconnected trigeminal nerve and facial parasympathetic outflow in the brainstem, the trigeminal-autonomic reflex has been proposed as one potential pathway of RES^4^. Trigeminovascular activation via the auriculotemporal nerve has also been proposed as an underlying mechanism of action^8^. The cervical-autonomic reflex was furthermore described as a contributor for the auricular-vascular response in upper cervical disorders via the second and third cervical roots, contributing to ear lobe innervation^8^. It has been theorized that the RES may result in conjunction with TACs, which may facilitate an imbalanced autonomic system to induce an inhibition of the sympathetic tone of the ear. The sympathetic dysregulation but not the parasympathetic activation is considered to be the predominant mechanism for the RES; therefore, the TAC is thought to play a minor role in isolated cases of RES^5^. These theories concern the trigemino-autonomic dysregulation in RES, TACs and associated clinical conditions, emphasizing inconsistency regarding the present knowledge on how the human auricle and its vasculature are innervated. Migraine can be associated with RES and is partially provoked by parasympathetic hyper-activation. Consequently, RES may also be a specific sign of parasympathetic activation during migraine^16^. Detailed information on the perivascular innervation of the human ear with particular focus on the auricular helix is helpful to clarify the underlying mechanisms causing RES, as an insight into autonomic alteration, e.g. as migraine.

This study **primarily** aimed to investigate the autonomic innervation of the auricular helix with particular focus on the perivascular zone using the co-transmitters predominantly for autonomic nerve supply for vasomotor control to identify the contributing axons in the context of sympathetic and parasympathetic innervation. A **second aim** was to use the unique approach combining three-dimensional (3D) reconstructions from human auricles cleared with the Spalteholz technique to clarify the consistency of the ear vasculature tree in addition to provide a three-dimensional map of the auricular arterial system. This may be considered as an important pathway for the distribution of sympathetic nerves in the ear in the context of the sympathetic nerves following the facial and ear arteries.

## Materials and Methods

While alive, the body donors gave written and informed consent to the donation of their tissues for research and teaching purposes. All tissues were obtained in accordance to the Death and Funeral Act of Vienna (version 2013, section 2, §12,13) and the Saxonian Death and Funeral Act (version 2014, section 8, §18). Ten paired auricles were removed from five human cadavers in a fresh and anatomically unfixed condition (mean age 83.4 ± 10.3 years, 3 females and 2 males) from the Medical University of Vienna. Another two auricles were obtained from a 96-year-old female cadaver from the University of Leipzig. Institutional approval was obtained from both Universities according to this legislation.

### Spalteholz technique with consecutive vascular staining and *μ*-CT

Ten paired auricles were rinsed in isotonic saline prior to injecting red silicone (Elastosil RT 601, Wacker Chemie AG, Burghausen, Germany), according to the procedure shown by Zilinsky *et al*.^2^. After casting in resin, the auricles were fixed in 10% (by volume) formaldehyde for three days, then rinsed in tap water and 4% (by volume) hydrogen peroxide for one day each, and then dehydrated in ascending ethanol for five days. Following this, the auricles were transferred into wintergreen oil (methyl ester of salicylic acid, Sigma-Aldrich, Munich, Germany) for two days before being placed in a fresh bath of wintergreen oil, as described by Spalteholz^17^.

Following the injection procedure, the auricles were scanned using a General Electric phoenix v/tome/x s *μ*-CT (GE Measurement & Control Solutions, Wunstorf, Germany) with an isovoxel size of 40 (± 2) *μ*m. Segmentation of the region of interest was carried out using Software VG Studio MAX 3.0 (Volumen Graphics GmbH, Heidelberg, Germany), with a threshold-based separation of soft tissue from the vascular injection agent. Larger artifacts were removed manually prior to importing the data as STL files in 3-matic (Materialise GmbH, Gilching, Germany) and then exporting as a 3D-PDF. These files served as a basis to analyze the distribution of the helical arcade and helical rim arcade.

### Histology and Immunohistochemistry

**Figure 1:**
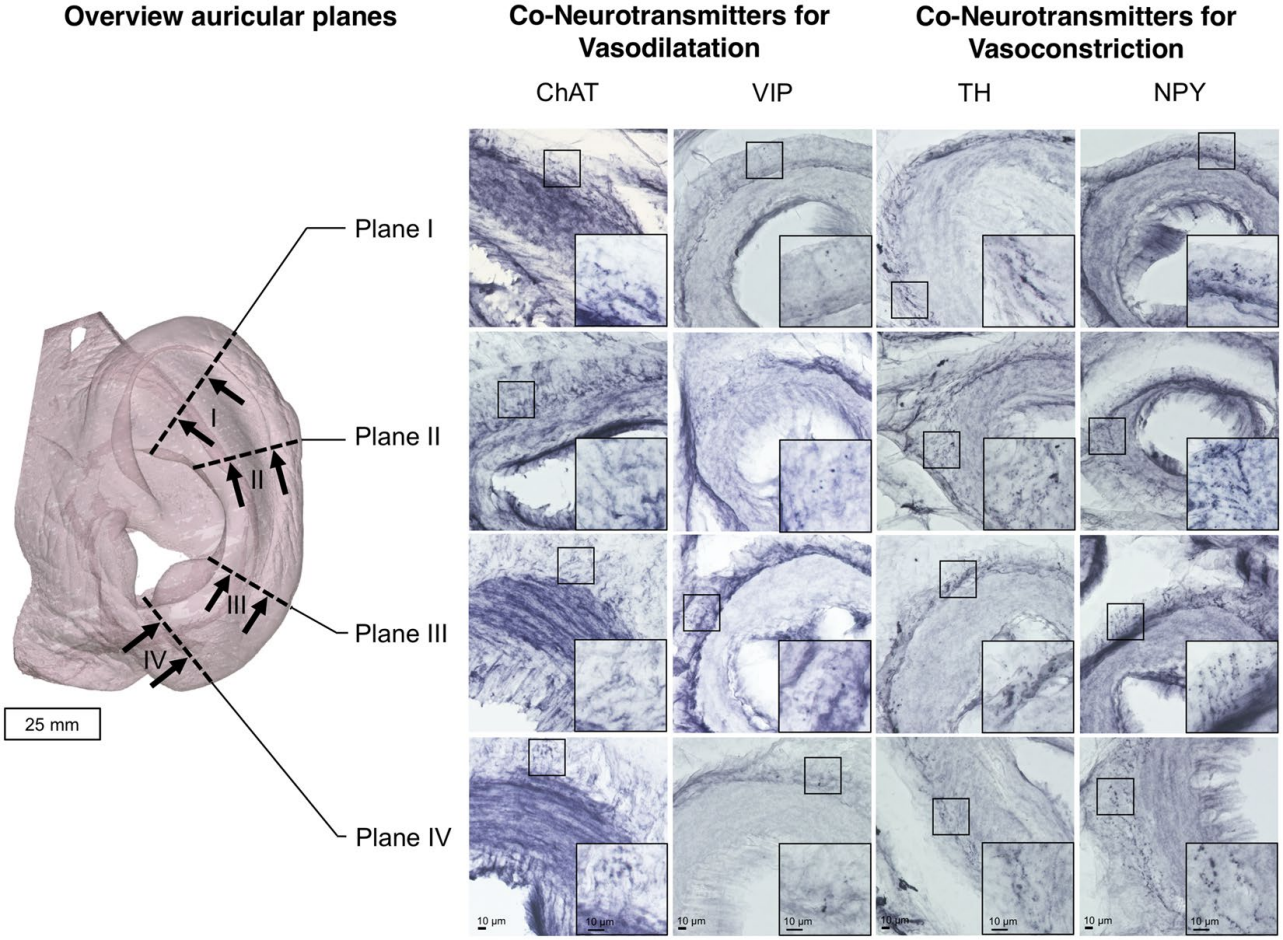
Magnification of a Spalteholz ear showing the area four sections which were used for histology and immunohistochemistry (left), and Anti-choline acetyltransferase (ChAT), anti-vasoactive intestinal peptide (VIP) stainings of the perivascular region indicating cholinergic/parasympathetic innervation as well as well as anti-tyrosine hydroxylase (TH) and anti-neuropeptide Y (NPY) stainings of the perivascular region indicating sympathetic innervation.

Immediately after removal two auricles were immersion-fixed in 4% paraformaldehyde (PFA) with phosphate buffer saline (PBS; 0.1 M; pH 7.4) for six weeks. One ear was cut into radial segments as shown in Fig. 1. Planes were chosen with the external acoustic meatus as an axis. For cryoprotection, segments were immersed in 30% sucrose in PBS with 0.1% sodium azide. 30 µm thick cryosections were made on a cryomicrotome Zeiss Hyrax S30 with freezing unit Zeiss hyrax KS34; sections were collected in PBS with 0.1% sodium azide.

For the hematoxylin-eosin staining, sections were mounted on glass slides, dried and processed according to standard procedures^18^. Prior to immunohistochemical staining, further sections were treated with 60% methanol and 2% H_2_O_2_ for 1 hour, followed by a blocking step (blocking solution: PBS-T with 2% bovine serum albumin, 0.3% milk powder and 0.5% donkey serum) for one hour and washed in PBS-T (PBS with 0.05% Tween 20). Slices were incubated with the primary antibodies (diluted in blocking solution) overnight at 4 °C. Primary antibodies are listed in Table 1. After washing in PBS-T, the sections were incubated with biotinylated secondary antibodies (see Table 2) for one hour, followed by incubation with extravidin-peroxidase (Sigma, 1:2000) for one hour and visualization by a nickel-enhanced peroxidase reaction with 3,3′-diaminobenzidine. Sections were mounted onto glass slides and cover slipped with Entellan^®^ in toluene.

**Table 1:** Primary antibodies.

Structure(s) of interest		Detected proteins	Antibodies	Dilution	Source	Code
	Basal laminae	Laminin	Rabbit anti-Laminin	1:1500	DAKO	Z0097
Perivascular co-transmitters for vasoconstriction (sympathetic)	Noradrenergic terminals	Tyrosine hydroxylase (TH)	Mouse anti-TH	1:100	Chemicon/Millipore	MAB318
	Neuronal terminals	Neuropeptide Y (NPY)	Rabbit anti-NPY	1:1000	ImmunoStar	22840
Perivascular co-transmitters for vasodilatation (cholinergic/parasympathetic)	Neuronal terminals	Vasoactive intestinal peptide (VIP)	Rabbit anti-VIP	1:1500	ImmunoStar	20077
	Cholinergic terminals	Choline acetyl transferase (ChAT)	Goat anti-ChAT	1:400	Chemicon	AB144
	Synaptic contacts	Synaptophysin	Rabbit anti-synaptophysin	1:1000	DAKO	A010

**Table 2:** Secondary antibodies.

Antibody	Dilution	Source	Code
Biotinylated donkey anti-mouse	1:1000	Dianova	715-065-150
Biotinylated donkey anti-rabbit	1:1000	Dianova	711-065-152
Biotinylated donkey anti-goat	1:1000	Dianova	705-065-147

### Microscopy

Tissue sections were imaged using a Keyence research microscope (BZ9000, Keyence, Neu-Isenburg, Germany). Photoshop CS2 (Adobe Systems, Mountain View, CA, USA) was used to process the images with minimal alterations to color, saturation, contrast and background.

## Results

### Combined Spalteholz technique and *μ*-CT show the three-dimensional arrangement of the vascular supply of the auricle as being highly consistent

**Figure 2:**
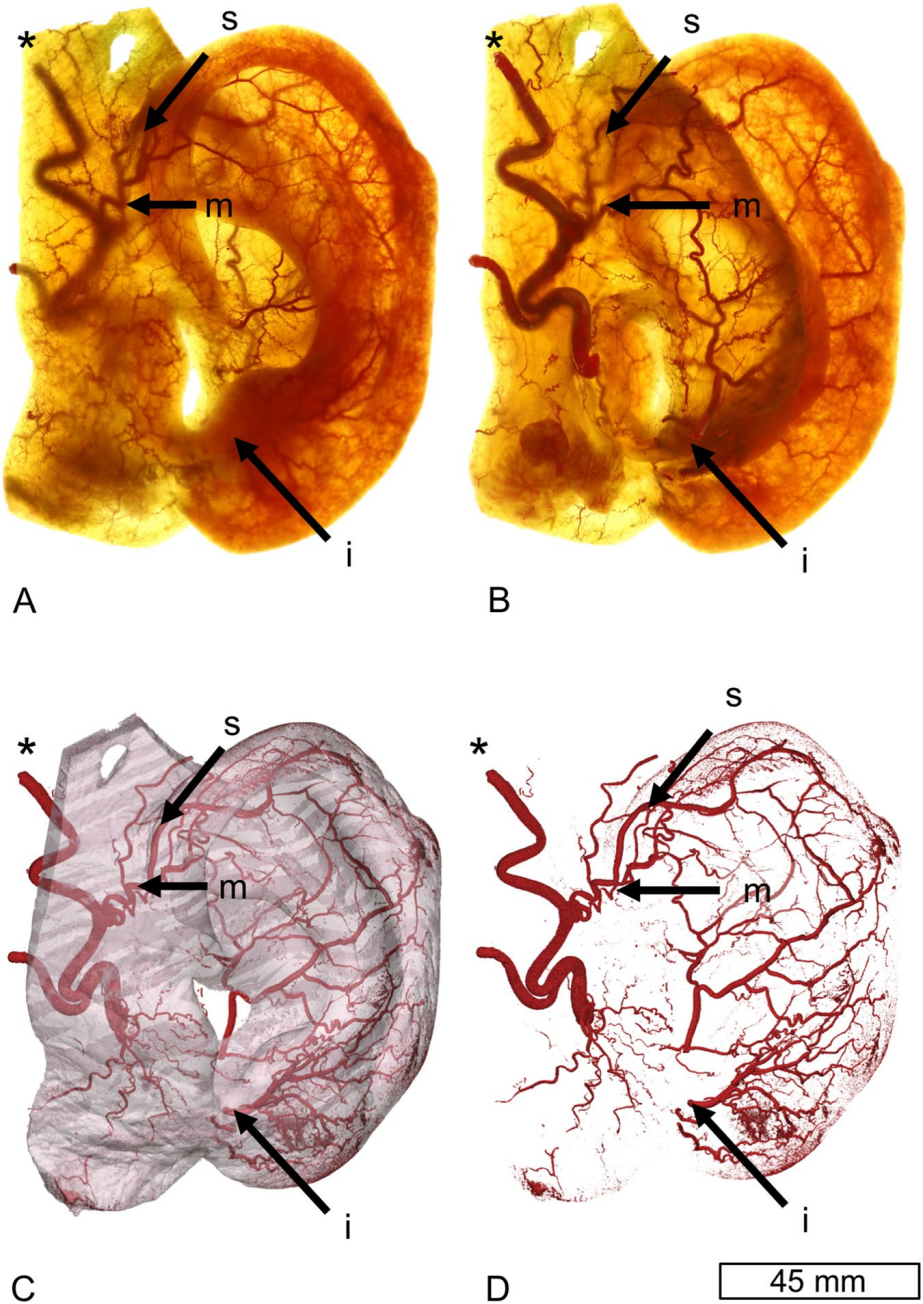
Lateral (**A**) and posterior view of a Spalteholz ear (**B**) showing auricular vascularity, and three-dimensional reconstruction using micro computed tomography in the surface topography (**C**) and isolated vascularity (**D**). s = superior anterior auricular artery, m = middle anterior auricular artery, i = inferior anterior auricular artery, *superficial temporal artery.

The *μ*-CT based reconstruction of the Spalteholz auricles has shown one to three distinct arches forming the helical rim arcade with contributions from the auricular branches, commonly with at least one or more continuous and one discontinuous arch, as shown in Fig. 2.

Three auricular branches were found frequently, originating from the superficial temporal artery (STA) and posterior auricular artery (PAA), contributing to the helical rim arcade. These vessels have been named as the superior, middle and inferior anterior auricular arteries. The STA was the main contributor to the superior anterior auricular artery in 7/10 cases (PAA in 3/10 cases) and the PAA was the main contributor to the middle and inferior anterior auricular artery in 9/10 and 10/10 cases, respectively. Similarly, the helical arcade was formed by one continuous arch (8/10 cases) with anastomoses to the helical rim arcade and predominantly superior and middle anterior auricular arteries forming an extended vascular network.

Although some variability exists regarding their feeders, namely the anterior auricular arteries, the helix is rich in supply with a few redundant arches, provided by the PAA and STA. One or more anastomosing arches were observed, and the number in these given experiments might be underestimated.

### Combined autonomic innervation in proximity to the helical arcade and rim arcade

A number of vessels were observed microscopically in those planes. Choline acetyl transferase positive (ChAT+) terminals were found in proximity to the vessels with increased density in planes II and III compared to planes II and III (Fig. 1). Vasoactive intestinal peptide positive (VIP+) innervation was observed in all planes without marked differences in density (Fig. 1). Equally, tyrosine hydroxylase positive (TH+) noradrenergic terminals and neuropeptide Y positive (NPY+) endings were observed throughout the helix, especially in planes I, II, and III, with increased density in planes I and III for NPY (Fig. 1).

The Hematoxylin-Eosin stained images gave an overview of the planes being observed (Supplement Fig. 1, left column). In proximity to the laminin-positive vasculature throughout the planes (Supplement Fig. 1, middle column), nerve fiber terminals were seen, indicated by synaptophysin staining (Supplement Fig. 1, right column).

## Discussion

This study, for the first time, presents data on the periarterial autonomic innervation of the human auricle in conjunction with a 3D visualization of the vascular tree, showing the proximity of autonomic nerve fibers to the perivascular beds of the helical arteries. It used a combination of the Spalteholz technique and *μ*-CT to depict auricular vascularity in detail in combination with histology and immunohistochemistry. Though the autonomic innervation of human ear has been investigated with both functional and surgical methodology^3–5,7–15,19–24^, this was to date not the case for the periarterial autonomic innervation.

In this given study, NPY+ nerve endings were observed in the perivascular zones throughout the auricular helix, consistent with animal work^25^. NPY is known to have a role as a vasoconstrictor^25^ and has been found as a neurotransmitter after ATP, co-released from sympathetic terminals^26^. Evidence also exists of co-storage of noradrenalin (NA) as a co-transmitter of NPY to potentiate the contractile response of vascular smooth muscle^25^, co-localized with TH throughout the arterioles in rats^27^ and in sympathetic TH+ neuron subpopulations guinea pig ear vessels^28^. The proposed key role of NPY is to potentiate the vasoconstrictor effects by noradrenaline^25,29,30^ with NPY+ neuron-induced vasoconstriction having longer effects than NA-induced constriction^31^. Combined NPY and TH immunostaining showed that they co-exist in perivascular sympathetic nerves^13–15^, as NPY has, to date, only been reported to be secreted by parasympathetic cranial nerve ganglia in glands but not in postganglionic perivascular terminals^22^. The simultaneous existence of NPY+ and TH+ terminals shown here indicates that the vasoconstriction of the auricular arteries is likely under the influence of sympathetic fibers via NPY and NA. In contrast to this evidence for vasoconstriction, driven by sympathetic innervation, the mechanism for vasodilatation of the ear is more complex. One theory for the ear skin is passive vasodilatation, i.e. the inhibition of the sympathetic vasoconstrictor fibers as opposed to activation of the parasympathetic fibers evoking dilation of the vasculature^3^.

In the current study, cholinergic co-neurotransmitters in the perivascular zone of the human auricle for active vasodilatation were shown for the first time. Acetylcholine (ACh) and VIP are both involved in early and late vasodilatation response as part of the parasympathetic system^32,33^. The release of ACh exists at perivascular nerves, with evidence for its function and presence in parasympathetic nerves in other regions^32,34,35^. The challenge to provide evidence for the latter forms the ambiguity regarding parasympathetic involvement in vasomotor control. While parasympathetic nerves play an active role in brain vasodilatation^36^, they may have a minute role elsewhere^34,37–40^. The lack of suitable markers to specify parasympathetic perivascular fibers adds even more complexity, and a potential reason is also the difficulty in interpreting immunological studies of parasympathetic innervation at the perivascular zone such as VIP^41^. VIP is a marker for parasympathetic nerves and a proof of parasympathetic contribution^4^. It has also been reported to be associated with non-cholinergic nerves. Another challenge for determining parasympathetic effects on vasodilatation is the cholinergic vasodilatation mediated by the sympathetic system^41–43^. Up to 15% of the paravertebral sympathetic ganglia have cholinergic neurons^44^, and cholinergic sympathetic vasodilatator nerves are involved in the cutaneous vasodilatation in humans^42,45^. The trigemino-facial reflex via the facial nerve parasympathetic fibers may cause vasodilatation of human red ear syndrome^4,46^, but clear evidence is lacking. Of interest is that the cranial vagus nerve terminals distribute to the ear via an auricular branch using two different pathways; one following the external ear canal and distributes predominantly to the cymba concha area of the auricular skin, and the other hijacking the posterior auricular branch of the facial nerve^23,24,47^. Though the facial and vagus nerve cholinergic contributions have territories in the human ear, they are unlikely to play a role in perivascular cholinergic fibers except for the greater auricular nerve. The greater auricular nerve with its cervical roots at the C2-C3^48^ may be an efferent arch of auricular vasodilatation response in both the trigeminal-autonomic reflex pathway and upper cervical disorders. This however remains to be substantiated in humans. Another contradiction of passive vasodilatation at the human ear is the difference in vasomotor control of glabrous and non-glabrous skin^49^. While NA+ fibers innervate the arterioles in glabrous zones of palms, cholinergic perivascular nerve fibers are present in the non-glabrous areas in human skin^49–54^. VIP may play a role in active vasodilatation of non-glabrous human skin^42^. Moreover, in the non-glabrous skin, as in the ear, VIP and ACh are co-transmitters of cholinergic nerve and they are described as the sympathetic cholinergic active vasodilatator system of the skin^55^. Based on the co-release of VIP and ACh in the periauricular zone in this study, it can be concluded that sympathetic cholinergic fibers conveying via greater auricular nerve are likely to provide the fibers driving the active vasodilatation of the human ear.

The STA and the PAA both originate from the external carotid artery and form important vessels of the viscerocranium. The sympathetic fibers for both arteries originate from the C2 to T1 (first thoracic segment)^56^, which is consistent with RES, which has been reported in migraine cases and upper cervical disorders, including C2 and greater auricular nerve anti-dromic vasodilatation studies^8,48^. For the trigemino-cervical complex the nociceptive afferents reside in the caudal region of the trigeminal nucleus caudalis and extend into the dorsal horns of C1 and C2^57^. The convergence of the trigeminal system with C2 and C3 can also explain the underlying mechanism of the non-cervical but cranially originated nociceptive disorders on auricular flashing episodes. The highly consistent three-dimensional distribution of the ear arteries was observed for the helical rim arcade and the helical arcade shown here is in line with previous anatomical and surgical studies on the blood supply^1,2,58^.

Auricular electrostimulation as a neuromodulation modality has been reported to alleviate pain syndromes including migraine^59^. The underlying mechanism of auricular electrostimulation is mainly attributed to the stimulation of the auricular branch of the vagus nerve in the context of auricular electrode placement^59^. As a consequence, the interactions between the trigeminal spinal nucleus and nucleus tractus solitarius are considered to play a key role in modulating the trigemino-autonomic dysregulation of both migraine and TACs. While the exact mechanism and contributing neural networks and reflex arches still need to be investigated in more detail, peri-arterial autonomic innervation of the auricles should be considered while interpreting the contributing autonomic pathways for auricular electrostimulation studies in the context of presented results.

A number of limitations exist for the given study. First, the sample size was small. Further advancements of the Spalteholz method exist, including CLARITY for three-dimensional histological assessment^60^, but have been untested to date for specimens such as full auricles. The number of anastomoses may have been underestimated as a consequence of blood clotting. Further differentiation of the origin of the perivascular cholinergic nerves and further studies to clarify the origin of these nerves are also pending.

The present study focused on the autonomic innervation of the periarterial tree of the human auricle and our results did not exclude any potential contributions of sensory nerve endings for vasodilation, such as the trigeminal nerve. The trigeminal nerve or free nerve endings may potentially contribute to auricular vasodilatation with the secretion of substance P and calcitonin gene-related peptide (CGRP) in humans^61,62^. Of relevance to migraine, a study reported elevated levels of CGRP in the jugular vein but not in the cubital vein during a migraine attack, which can be considered as a sign for local effects^63,64^. Another study, however, failed confirming such change in both veins^65^.

It is worth noting that substance P can induce both vasodilation and plasma protein extravasation, whereas the CGRP solely induces vasodilation, without known plasma extravasation effects in humans^66^. It can therefore be theorized that CGRP would be more likely to contribute auricular vasodilation and RES in migraine if any contributions may exist via trigeminal nerve and/or free nerve endings. To date, the presence of CGRP and its role as a vasodilatator has been reported in human skin, meningeal vascular smooth muscle cells and rat ear skin^67–70^. Further studies are needed to investigate this role on human auricular vasomotor control with interactions of autonomic vasomotor neurotransmitters, which have been demonstrated in the given study.

## Conclusions

This study gave evidence for ChAT+ and VIP+ nerve terminals along with NPY+ and TH+ terminals at the human auricular helix, indicating the innervation of active vasoconstriction and -dilatation. These terminals were found in the perivascular zone, formed by terminal branches of the superficial temporal artery and posterior auricular artery. The 3D reconstruction from *μ*-CT of ears showed a highly consistent pattern of anterior auricular branches, potentially forming the pathways for the perivascular autonomic nerve system.

## Electronic supplementary material

Supplement figure 1
