# Supplementary material for: Peri-arterial Autonomic Innervation of the Human Ear
Source: Sci Rep. 2018 Jul 31;8:11469. doi: 10.1038/s41598-018-29839-z (PMC6068185; doi:10.1038/s41598-018-29839-z)

Peri-arterial Autonomic Innervation of the Human Ear

Cakmak, Yusuf Ozgur [1], Cotofana, Sebastian [2], Jäger, Carsten [3], Morawski, Markus [3], Sora, Mircea-Constantin [4,5], Werner, Michael [6], Hammer, Niels [1,6,7]

1 University of Otago, Department of Anatomy, Dunedin, New Zealand

2 Albany Medical College, Department of Anatomy, Albany, NY, USA

3 University of Leipzig, Paul-Flechsig-Institute for Brain Research, Leipzig, Germany

4 Sigmund-Freud Private University Vienna, Centre for Anatomy and Molecular Medicine, Vienna, Austria

5 Medical University of Vienna, Zentrum für Anatomie und Zellbiologie, Vienna, Austria

6 Fraunhofer Institute for Machine Tools and Forming Technology, Dresden, Germany

7 Department of Trauma, Orthopedic and Plastic Surgery, University Hospital of Leipzig, Germany

Corresponding author

Niels Hammer, M.D., Department of Anatomy, University of Otago, Lindo Ferguson Building, 270 Great King St, Dunedin 9016, New Zealand; Phone: +64 3 479 7362, Fax: +64 3 479 7254,

Email: nlshammer@googlemail.com

Supplement figure 1: Hematoxylin-eosin stained (H&E) ear samples (left column, A), and anti-laminin (middle column, A) and –synaptophysin (right column, C) stainings of the perivascular region.

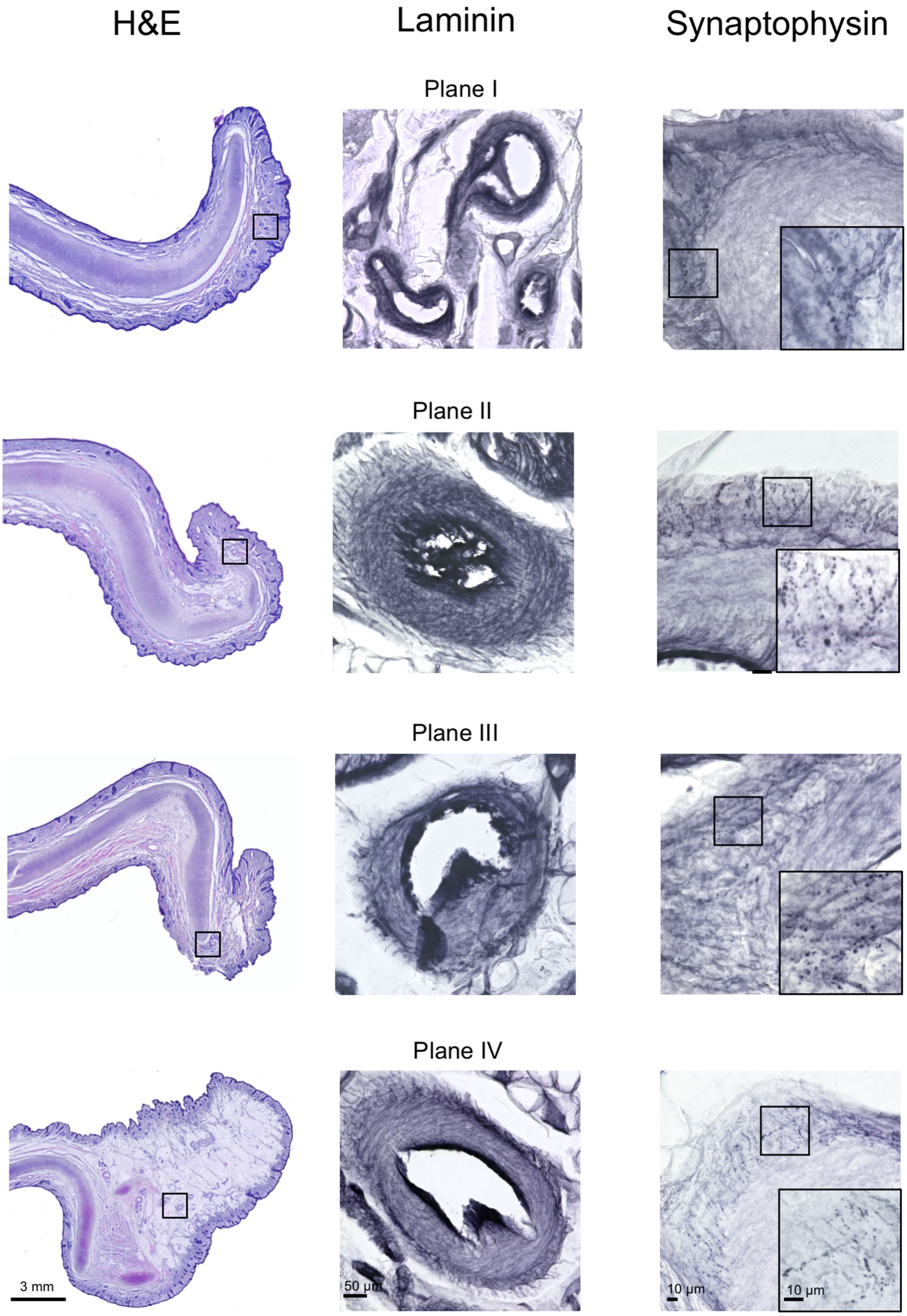

Supplement: Supplementary file 1 — Supplement figure 1 [file 41598_2018_29839_MOESM1_ESM.pdf]
